# Quantitative Benefit–Risk Evaluation of Rivaroxaban in Patients After Peripheral Arterial Revascularization: The VOYAGER PAD Trial

**DOI:** 10.1161/JAHA.123.032782

**Published:** 2024-04-02

**Authors:** Zhong Yuan, Bennett Levitan, Hsiaowei Deng, Michael Szarek, Rupert M. Bauersachs, Scott D. Berkowitz, Lloyd Haskell, Elliot S. Barnathan, Marc P. Bonaca

**Affiliations:** ^1^ Janssen Research & Development LLC Horsham PA; ^2^ Janssen Research & Development LLC Titusville NJ; ^3^ CPC Clinical Research, Department of Medicine University of Colorado Aurora CO; ^4^ Cardioangiologisches Centrum Bethanien ‐ CCB Gefäß‐Centrum Frankfurt am Main Germany

**Keywords:** health state utilities, modeling, multi‐criteria decision analysis, peripheral artery disease, rivaroxaban, Vascular Disease

## Abstract

**Background:**

The VOYAGER PAD (Efficacy and Safety of Rivaroxaban in Reducing the Risk of Major Thrombotic Vascular Events in Subjects With Symptomatic Peripheral Artery Disease Undergoing Peripheral Revascularization Procedures of the Lower Extremities) trial compared rivaroxaban (2.5 mg twice a day) plus aspirin with aspirin alone in patients with symptomatic peripheral artery disease requiring endovascular or surgical limb revascularization, with 50% receiving clopidogrel background therapy. The New Drug Indication application includes benefit–risk assessments using clinical judgment to balance benefits against risks. During its review, the US Food and Drug Administration requested additional quantitative benefit–risk analyses with formal weighting approaches.

**Methods and Results:**

Benefits and risks were assessed using rate differences between treatment groups (unweighted analysis). To account for clinical importance of the end points, a multi‐criteria decision analysis was conducted using health state utility values as weights. Monte Carlo simulations incorporated statistical uncertainties of the event rates and utility weights. Intent‐to‐treat and on‐treatment analyses were conducted. For unweighted intent‐to‐treat analyses, rivaroxaban plus aspirin would result in 120 (95% CI, −208 to −32) fewer events of the primary composite end point (per 10 000 patient‐years) compared with aspirin alone. Rivaroxaban caused an excess of 40 (95% CI, 8–72) Thrombolysis in Myocardial Infarction major bleeding events, which was largely driven by nonfatal, nonintracranial hemorrhage Thrombolysis in Myocardial Infarction major bleeding events. For weighted analyses, rivaroxaban resulted in the utility equivalent of 13.7 (95% CI, −85.3 to 52.6) and 68.1 (95% CI, 7.9–135.7) fewer deaths per 10 000 patient‐years (intent‐to‐treat and on‐treatment, respectively), corresponding to probabilities of 64.4% and 98.7%, respectively, that benefits outweigh risks favoring rivaroxaban per Monte Carlo simulation.

**Conclusions:**

These analyses show a favorable benefit–risk profile of rivaroxaban therapy in the VOYAGER PAD trial, with findings generally consistent between the unweighted and weighted approaches.

**Nonstandard Abbreviations and Acronyms**
ALI: acute limb ischemia
ATLAS II: An Efficacy and Safety Study for Rivaroxaban in Patients With Acute Coronary Syndrome
B‐R: benefit–risk
COMMANDER HF: A Study to Assess the Effectiveness and Safety of Rivaroxaban in Reducing the Risk of Death, Myocardial Infarction or Stroke in Participants With Heart Failure and Coronary Artery Disease Following an Episode of Decompensated Heart Failure
COMPASS: Cardiovascular Outcomes for People Using Anticoagulation Strategies
FDA: US Food and Drug Administration
HSUV: health state utility value
KM: Kaplan–Meier
MACE: major adverse cardiovascular events
MCDA: multi‐criteria decision analysis
TIMI: Thrombolysis in Myocardial Infarction
Tufts‐CEVR: Tufts Center for Evaluation of Value and Risk in Health
VOYAGER PAD: Efficacy and Safety of Rivaroxaban in Reducing the Risk of Major Thrombotic Vascular Events in Subjects With Symptomatic Peripheral Artery Disease Undergoing Peripheral Revascularization Procedures of the Lower Extremities

**Clinical Perspective**

**What Is New?**

- The investigation of a medicinal product often entails a wide spectrum of efficacy and safety end points that have different clinical consequences, which may pose challenges to benefit–risk assessment.
- We conducted a structured, unweighted benefit–risk evaluation using between‐treatment rate differences to quantify the benefit–risk balance of rivaroxaban therapy of the VOYAGER PAD (Efficacy and Safety of Rivaroxaban in Reducing the Risk of Major Thrombotic Vascular Events in Subjects With Symptomatic Peripheral Artery Disease Undergoing Peripheral Revascularization Procedures of the Lower Extremities) trial, as well as multi‐criteria decision analyses using health state utility values as weights to consider the collective clinical consequences of the study end points.

**What Are the Clinical Implications?**

- Structured benefit–risk assessment methods can help clinicians easily quantify and better understand the favorable benefit–risk profile of rivaroxaban plus aspirin therapy compared with aspirin alone in the VOYAGER PAD trial.
- Results showed that benefits outweigh risks favoring rivaroxaban. The generally consistent findings between the unweighted and weighted approaches reinforce the robustness of the benefit–risk results.

Peripheral artery disease (PAD) is characterized by the accumulation of atherosclerotic plaque in noncoronary arteries and generally refers to disease in the lower extremities. Adverse outcomes are driven by progressive atherosclerosis and related thrombosis in major arteries associated with underlying atheroma and chronic inflammation.^1^ Worldwide, PAD is the third leading cause of atherosclerotic vascular morbidity after coronary artery disease and stroke; notably, patients with PAD are at high risk of major adverse cardiovascular events (MACE) and major adverse limb events. Furthermore, the estimated prevalence of PAD appears to be increasing, with >200 million people living with PAD globally, including >40 million in Europe and >14 million in North and South America.^2^ In the United States alone, >8.5 million individuals 40 years and older are estimated to be affected by PAD.^3^, ^4^

Patients with an advanced stage of PAD are at elevated risk for limb events, particularly acute limb ischemia (ALI).^5^, ^6^ Revascularization is often indicated based on symptoms and progression of the disease with a high subsequent risk of MACE, major amputation, and all‐cause death.^7^ Patients with ALI require emergency endovascular or surgical peripheral revascularization, whereas elective peripheral revascularization is considered in patients with critical limb ischemia, depending on symptoms, including severity of intermittent claudication, pain at rest, or tissue loss.^8^ However, despite surgical interventions and currently available antithrombotic treatments, the risk of MACE and major adverse limb events following revascularization remains elevated.^9^, ^10^

Guidelines recommend single antiplatelet therapy for secondary prevention of thrombotic events in patients with PAD, dual antiplatelet therapy for patients with PAD after endovascular revascularization, and long‐term single antiplatelet therapy in all patients with PAD. Recommendations of dual antiplatelet therapy (clopidogrel and aspirin) after peripheral revascularization are class II,^11^, ^12^ although it was noted in a 2016 American Heart Association/American College of Cardiology guideline that the effectiveness of dual antiplatelet therapy (clopidogrel and aspirin) to reduce the risk of cardiovascular ischemic events in patients with symptomatic PAD is not well established.^11^ The clinical efficacy and safety of direct oral anticoagulants were not fully investigated or established until the COMPASS (Cardiovascular Outcomes for People Using Anticoagulation Strategies) trial.^13^ In a subgroup of patients with PAD, rivaroxaban 2.5 mg twice daily in combination with aspirin reduced MACE relative to aspirin alone.^14^ However, the data associated with revascularization in this subpopulation were limited.

The VOYAGER PAD (Efficacy and Safety of Rivaroxaban in Reducing the Risk of Major Thrombotic Vascular Events in Subjects With Symptomatic Peripheral Artery Disease Undergoing Peripheral Revascularization Procedures of the Lower Extremities) study was an international, multicenter, randomized, double‐blind, placebo‐controlled, phase 3 trial investigating the efficacy and safety of rivaroxaban to reduce the risk of major thrombotic vascular events in patients with symptomatic PAD undergoing lower extremity revascularization procedures.^15^ Patients were randomized to rivaroxaban 2.5 mg twice daily or placebo while on a background of low‐dose aspirin (100 mg daily),^16^ and, in an intent‐to‐treat (ITT) analysis, rivaroxaban significantly reduced the primary efficacy composite MACE plus major adverse limb events end point.^17^ Importantly, the rivaroxaban benefit was greater in a prespecified on‐treatment analysis. In the New Drug Indication Application, the sponsor included a prespecified benefit–risk (B‐R) assessment that used clinical judgment to balance benefits versus risks in these 2 analyses. During the review of the New Drug Indication application, the US Food and Drug Administration (FDA) requested the sponsor conduct additional quantitative B‐R analyses that included formal assessments of weights for each end point to address this difference and related issues. The current article summarizes results from an analysis conducted using the multi‐criteria decision analysis (MCDA) approach.

## Methods

### Data Sharing Statement

The data sharing policy of Janssen Pharmaceutical Companies of Johnson & Johnson (J&J) is available at https://www.janssen.com/clinical‐trials/transparency. The data supporting the findings of this study may be obtained from the authors, if the request is approvable per the J&J data sharing policy.

### Study Population

Participants in the VOYAGER PAD trial were previously described in detail (ClinicalTrials.gov identifier: NCT02504216).^16^, ^17^ In brief, participants who were 50 years and older, had symptomatic PAD, and underwent a successful revascularization procedure (endovascular [including hybrid] or surgical) for infrainguinal disease within the previous 10 days for PAD symptoms were randomly assigned in a 1:1 ratio to receive rivaroxaban at a dose of 2.5 mg twice daily or placebo, while taking a background of low‐dose aspirin (100 mg daily) with or without clopidogrel (at the treating team's discretion). The trial was event‐driven with a median follow‐up of approximately 31 months (interquartile range, 25–37 months).

The protocol was approved by the relevant ethics committee at each participating site and according to local regulations, and participants gave written informed consent. An independent data and safety monitoring committee monitored unblinded information for safety at specified intervals and performed 1 unblinded review for efficacy superiority as prespecified in the committee charter.

### Outcomes

The primary efficacy outcome was a composite of ALI, major amputation for vascular causes, myocardial infarction, ischemic stroke, or cardiovascular death. The primary safety outcome was major bleeding defined according to Thrombolysis in Myocardial Infarction (TIMI) classification: the composite of fatal bleeding, any intracranial bleeding, or clinically overt signs of hemorrhage associated with a drop in hemoglobin of ≥5 g/dL or a ≥15% absolute decrease in hematocrit. The events in both the primary efficacy outcome and the principal safety outcome vary considerably in their clinical impact, motivating the need to consider them individually in B‐R assessments.

**Figure 1:**
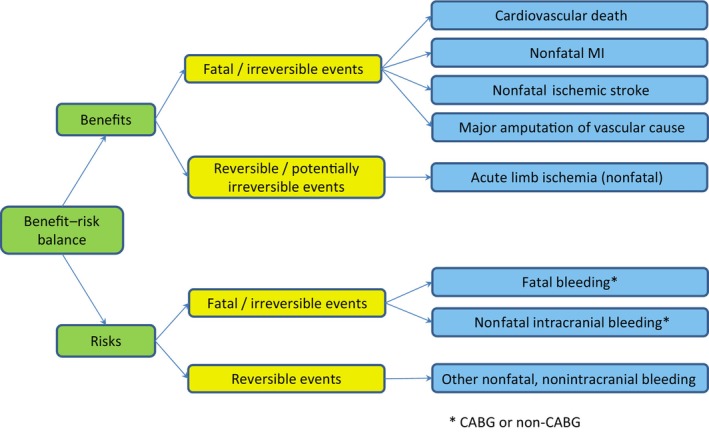
The value tree for VOYAGER PAD (Efficacy and Safety of Rivaroxaban in Reducing the Risk of Major Thrombotic Vascular Events in Subjects With Symptomatic Peripheral Artery Disease Undergoing Peripheral Revascularization Procedures of the Lower Extremities) benefit–risk assessment. CABG indicates coronary artery bypass graft; and MI, myocardial infarction.

The following key end points were identified for the B‐R assessment (Figure 1) using an approach that followed FDA draft guidance.^18^ Benefits include ischemic events, while risks include major bleeding events. Both benefit and risk end points were grouped by whether they were fatal/irreversible, a distinction used in the communication of the B‐R assessments by the FDA.^19^, ^20^ To avoid double‐counting deaths, end points other than cardiovascular death and fatal bleeding were defined as nonfatal.

### Statistical Analysis

B‐R analyses were conducted for efficacy and safety endpoints identified in the value tree based on randomized treatment assignment for the following analysis populations and data scopes:

- “ITT/until efficacy cutoff date” or ITT analysis: (1) the ITT analysis population included all unique randomized participants; and (2) the ITT data scope included all events from randomization until the study efficacy cutoff date, regardless of when they occurred relative to permanent treatment discontinuation.
- “Safety/on‐treatment” or on‐treatment analysis: (1) the safety analysis population included all unique randomized participants who took ≥1 dose of study drug; and (2) the on‐treatment data scope included all events from randomization until the last dose of study drug plus 2 days, excluding events and follow‐up >2 days after the last dose.

The B‐R assessment is considered exploratory with no a priori hypothesis testing and complementary to the efficacy and safety analyses previously reported.^17^

### Structured Unweighted B‐R Assessment

B‐R assessment entails quantifying benefits in the context of risks. However, comparing treatment hazard ratios (HRs) associated with benefits and risks is neither clinically intuitive nor conceptually straightforward because baseline event rates vary and the clinical consequences of these end points differ. In our analysis, the benefit and risk were assessed as rate differences (with 95% CIs) per 10 000 patient‐years (defined as the difference in incidence rate between the 2 treatments scaled to a hypothetical population of 10 000 patient‐years to reflect benefits and risks at a population level). These rate differences can be interpreted as the number of patients in this population who would experience a particular event when treated with rivaroxaban minus those in the same population treated with a placebo. The results were shown in forest plots, an approach often used in B‐R assessments^21^, ^22^, ^23^ to provide comprehensive visualization of the overall B‐R profile. This facilitates the reader's own interpretation of the results, particularly important fatal or irreversible events, as outlined in communications by the FDA.^19^, ^20^ The 95% CIs were calculated to demonstrate uncertainty only, and no hypothesis tests were conducted nor any adjustments for multiplicity applied.

To show the benefit and risk over time, the rate differences in Kaplan–Meier (KM) estimates between rivaroxaban and placebo were plotted for the primary efficacy composite end point versus the primary safety composite end point.

### Weighted B‐R Assessment Using the MCDA Approach

The MCDA approach is a well‐established, general decision analysis technique for solving complex decision problems that involve multiple outcomes or end points.^24^, ^25^, ^26^ Data S1 describes the steps in MCDA and key inputs are shown in Tables S1 through S4.

Two MCDA models were developed that included the same end points/outcomes except that model 1 included a composite of cardiovascular death and fatal TIMI major bleeding, whereas model 2 included all‐cause death (Table S1) for complete capturing of all death events. There were no clinically meaningful differences between the models, and the current report focuses on reporting the results of model 1.

A key element in MCDA is the application of weights that reflect the relative importance of different clinical end points. The Tufts Center for Evaluation of Value and Risk in Health (Tufts‐CEVR) was contracted to conduct an independent literature search to extract health state utility values (HSUVs) from the Tufts‐CEVR CEA (cost‐effectiveness analysis) registry, a database for cost‐utility analyses. A data set of 373 HSUVs was extracted, including major amputation (n=34), ALI (n=153), myocardial infarction (n=90), ischemic stroke (n=74), and intracranial (n=13) and nonintracranial (n=9) major bleeding. Of the 373 HSUVs, 159 were considered relevant primarily because of: (1) how closely the health state approximated end points in the VOYAGER PAD trial, and (2) how similar the assessment population was to that of VOYAGER PAD. Utility values for the relevant health states are presented in Table S2, and their interpretation as weights is given in Table S3. Because all end points are time‐to‐first‐event end points summarized/measured with incidence rates or KM rates, we used a modified version of MCDA that enables the MCDA score to be interpreted in units of deaths per 10 000 patient‐years. Data S1 describes the version of MCDA used in the present analyses, including the mathematical implementation of the weights. Table S4 provides the parameters used to do sensitivity analyses with the MCDA. In essence, when these HSUVs were applied, the analysis shows the MCDA score as the difference in the utility‐equivalent number of deaths between the treatment groups.

In addition to MCDA results using constant incidence rates, we assessed the temporal course of B‐R in the MCDA using KM rates.

## Results

In the VOYAGER‐PAD trial, 6564 patients were randomized to rivaroxaban (n=3286) or placebo (n=3278) and their baseline characteristics were well balanced (Table S5).^17^ Overall, patients had a median age of 67 years, 26% were women, 31% had symptomatic CAD, and the median ankle‐brachial index was 0.56. At randomization, nearly all patients were taking aspirin and 51% were taking clopidogrel.^17^ As previously shown in an ITT analysis, rivaroxaban 2.5 mg twice daily plus aspirin was associated with a significantly lower risk of developing a primary efficacy outcome event relative to aspirin alone (HR, 0.85 [95% CI, 0.76–0.96]), with nominal increases in TIMI major bleeding (HR, 1.43 [95% CI, 0.97–2.10]; on‐treatment analysis), cardiovascular death (HR, 1.14 [95% CI, 0.93–1.40]; ITT analysis), and all‐cause death (HR, 1.08 [95% CI, 0.92–1.27]; ITT analysis). The analysis below further quantifies the tradeoff between benefits and risks using an unweighted excess number of events and utility‐weighted MCDA scores, respectively.

### Structured Unweighted B‐R Assessment

**Figure 2:**
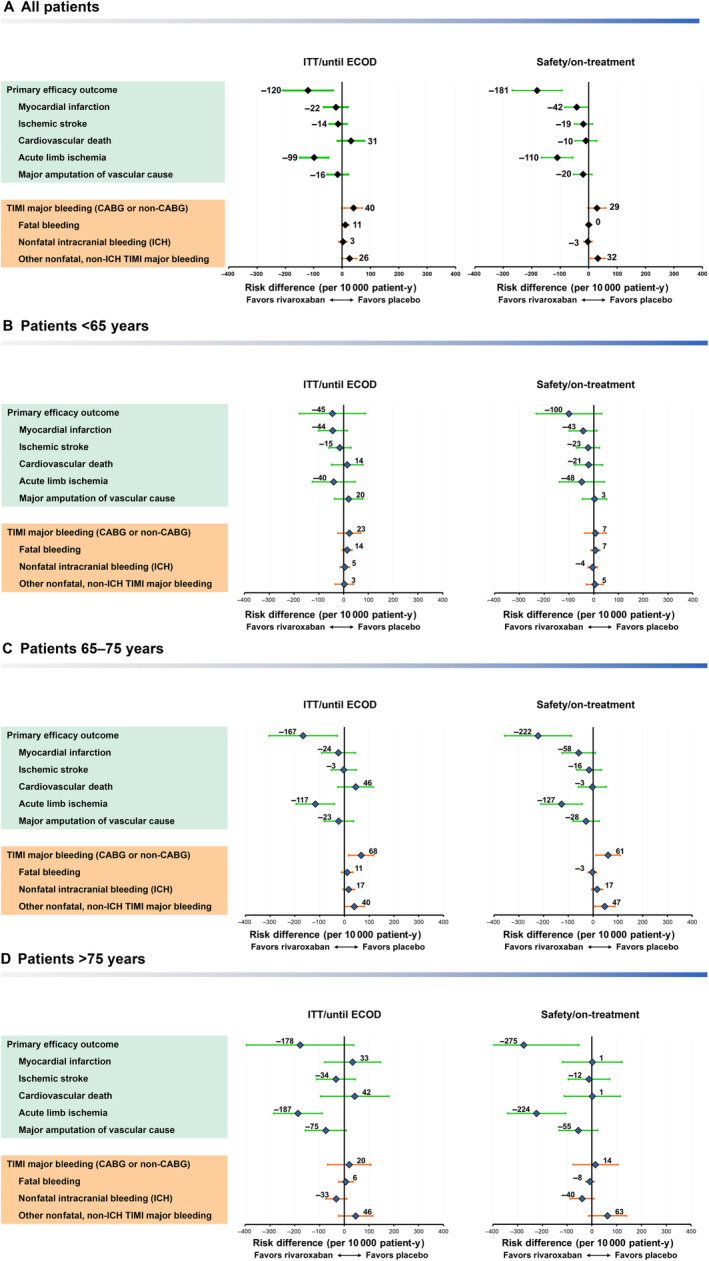
Forest plot showing excess number of patients with event for first occurrence of primary efficacy and safety outcomes and component events (rivaroxaban 2.5 mg twice daily plus aspirin 100 mg once daily vs placebo twice daily plus aspirin 100 mg once daily) incidence rate per 10 000 patient‐years in the intent‐to‐treat (ITT)/until efficacy cutoff date (ECOD) (left side) and safety/on‐treatment (right side) populations for all patients (A) and subgroups by age category: younger than 65 years (B), 65 to 75 years (C), and older than 75 years (D). “Other nonfatal, non‐ICH TIMI major bleedings” include clinically overt signs of hemorrhage associated with a drop in hemoglobin of ≥5 g/dL or a ≥15% absolute decrease in hematocrit. CABG indicates coronary artery bypass graft; cardiovascular death, death from cardiovascular causes; ICH, intracranial hemorrhage; and TIMI, Thrombolysis in Myocardial Infarction.

Based on ITT analyses, on average, treatment with rivaroxaban plus aspirin would result in 120 (95% CI, −208 to −32) fewer events of the primary composite end point (per 10 000 patient‐years) compared with placebo plus aspirin, which was largely driven by the reduction of ALI (99 fewer events [95% CI, −149 to −49]) but also showed a numeric increase of 31 (95% CI, –16 to 78) cardiovascular deaths (Figure 2). Rivaroxaban caused an excess of 40 (95% CI, 8–72) TIMI major bleeding events, which was largely driven by nonfatal, non−intracranial hemorrhage TIMI major bleeding. The B‐R profile was more favorable in on‐treatment analyses; treatment with rivaroxaban plus aspirin resulted in 181 (95% CI, −269 to −94) fewer primary efficacy events compared with placebo plus aspirin but caused an excess of 29 (95% CI, –2 to 60) TIMI major bleeding events (Figure 2). Generally consistent findings were observed among age subgroup categories, except for major amputation favoring placebo in patients younger than 65 years (Figure 2). In the on‐treatment analysis, all individual components of the primary efficacy end point contributed to the positive treatment effect, including cardiovascular death, with no imbalance in fatal or nonfatal intracranial hemorrhage. Generally consistent findings were observed among age subgroup categories, except for major amputation and fatal bleeding favoring placebo in patients younger than 65 years (Figure 2). As an alternative perspective on these rate differences, Table S6 provides the number needed to treat and number needed to harm for each end point in these analyses for all patients and the age subgroups.

**Figure 3:**
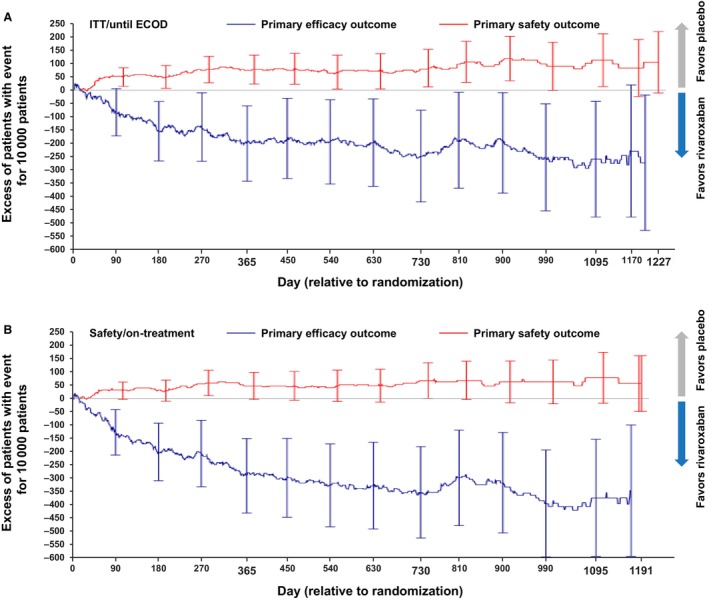
Kaplan–Meier cumulative incidence risk difference for the primary efficacy and safety outcomes (comparing rivaroxaban vs placebo). (**A**) Intent‐to‐treat (ITT)/until efficacy cutoff date (ECOD) and (**B**) safety/on‐treatment. *Rivaroxaban 2.5 mg twice daily plus aspirin 100 mg once daily vs placebo twice daily plus aspirin 100 mg once daily. The primary efficacy outcome is the composite of acute limb ischemia, major amputation for vascular causes, myocardial infarction, ischemic stroke, or cardiovascular death. The primary safety outcome is Thrombolysis in Myocardial Infarction major bleeding (primary safety variable). The x‐axis of the Kaplan–Meier figures is restricted to the period in which at least 10% of the analysis set patients are at risk as suggested by Pocock et al.^27^

For the treatment effect over time, the ITT and on‐treatment analysis results based on KM plots performed per Pocock et al^27^ (Figure 3) were generally consistent. The reduction of events in the primary efficacy end point with rivaroxaban plus aspirin started early and continued to improve throughout the course of treatment, with a greater effect seen for the on‐treatment analysis. The increase in TIMI major bleeding developed gradually and plateaued at ≈270 days, suggesting that, throughout the course of the study, the cumulative benefit would exceed the cumulative risk for rivaroxaban plus aspirin compared with placebo plus aspirin.

### Weighted B‐R Assessment Using the MCDA Approach

**Figure 4:**
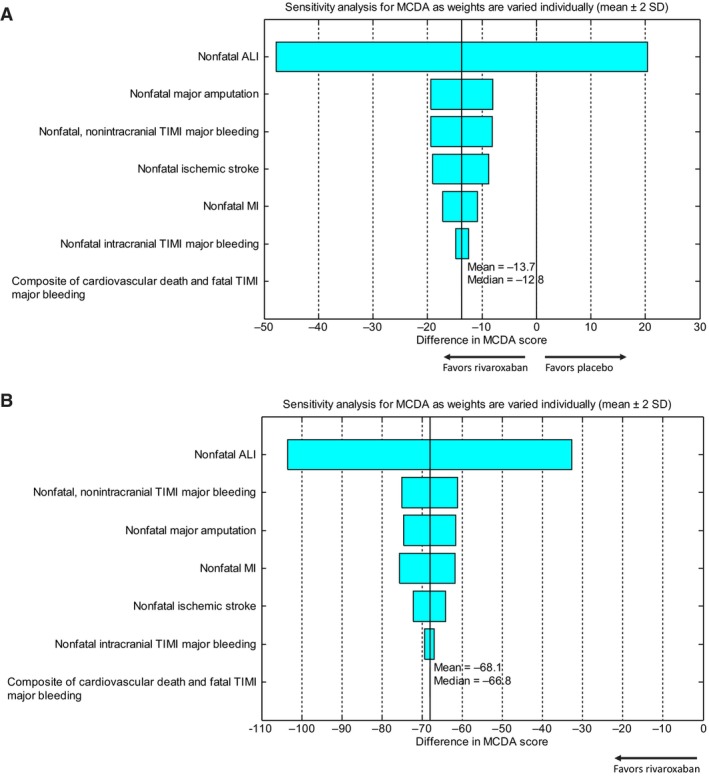
One‐way weight‐distribution sensitivity analysis (model 1, intent‐to‐treat/until efficacy cutoff date) (A) and 1‐way weight‐distribution sensitivity analysis (model 1, safety/on‐treatment) (B). ALI indicates acute limb ischemia; cardiovascular death, death from cardiovascular causes; MI, myocardial infarction; and TIMI, Thrombolysis in Myocardial Infarction. The solid black vertical line reflects the point estimate for the multi‐criteria decision analysis (MCDA) score difference. The width of the bars reflects the extent to which the MCDA score difference point estimate changes as the weight for each scenario is varied.

The MCDA results of model 1 in the ITT analysis are shown in Table 1. The difference in MCDA score between rivaroxaban plus aspirin and placebo plus aspirin was −13.7 (95% CI, −85.3, 52.6), where both the point estimate and 95% CI are based on a Monte Carlo simulation with 5 000 000 simulation runs. This point estimate suggests that treatment with rivaroxaban plus aspirin resulted in the utility equivalent of 13.7 fewer deaths per 10 000 patient‐years compared with placebo plus aspirin, with a 95% CI including 0. We also conducted a 1‐way weight distribution sensitivity analysis, in which the weight assigned to each nonfatal end point was changed by ±2 SDs from its mean (Table S2) while other weights were kept constant. The difference in MCDA score was computed over this range. A large change in the difference in MCDA score when varying the end point weight indicates a larger sensitivity of the MCDA results to that weight. Notably, the favorable B‐R balance associated with rivaroxaban was relatively insensitive to changes of utility values for all end points for the on‐treatment analysis but not for ALI in the ITT analysis (Figure 4).

**Table 1:** Clinical Outcomes, Weights, Contributions, and Differences in MCDA Score and Probability That Benefits Outweigh Risks (Model 1, ITT/Until ECOD and Safety/On‐Treatment Analyses)

Study end point* (ITT analysis)	Rivaroxaban (rate per 10 000 patient‐y)	Placebo (rate per 10 000 patient‐y)	Rate difference (95% CI)†	Mean weight (1 – utility)	Contribution (from Monte Carlo)‡ ^,^ §
Composite of cardiovascular death and fatal TIMI major bleeding	264.2	227.7	36.5 (−12.1 to 85.1)	1.00	36.5
Nonfatal major amputation of vascular cause	120.5	142.5	−21.9 (−57.9 to 14.1)	0.59	−12.9
Nonfatal, ICH TIMI major bleeding	22.7	20.2	2.5 (−11.9 to 17)	0.42	1.1
Nonfatal ALI	191.0	291.3	−100.3 (−149.6 to −51)	0.36	−36.1
Nonfatal ischemic stroke	76.3	90.4	−14.1 (−42.6 to 14.5)	0.35	−4.9
Nonfatal MI	141.0	154.3	−13.3 (−51.5 to 24.8)	0.22	−2.9
Nonfatal, non‐ICH TIMI major bleeding	77.6	51.9	25.7 (0.5–50.8)	0.22	5.6
Between‐treatment difference in MCDA score‖ (95% CI§) (utility death equivalents/10 000 patient‐y)				−13.7 (−85.3 to 52.6)	
Probability of benefits outweigh risks§				64.4%	

Study end point* (on‐treatment analysis)	Rivaroxaban (rate per 10 000 patient‐y)	Placebo (rate per 10 000 patient‐y)	Rate difference (95% CI)†	Mean weight (1 – utility)	Contribution (from Monte Carlo)‡
Composite of cardiovascular death and fatal TIMI major bleeding	127.3	138.6	−11.4 (−50.8 to 28)	1.00	−11.4
Nonfatal major amputation of vascular cause	74.4	99.5	−25.1 (−57.2 to 7)	0.59	−14.8
Nonfatal, ICH TIMI major bleeding	16.9	19.7	−2.7 (−17.4 to 11.9)	0.42	−1.2
Nonfatal ALI	189.6	293.7	−104.1 (−158 to −50.2)	0.36	−37.5
Nonfatal ischemic stroke	69.4	80.5	−11 (−40.7 to 18.6)	0.35	−3.9
Nonfatal MI	113.0	141.9	−28.9 (−67.7 to 9.9)	0.22	−6.4
Nonfatal, non‐ICH TIMI major bleeding	69.4	37.9	31.5 (6.4–56.7)	0.22	6.9
Between‐treatment difference in MCDA score‖ (95% CI§) (utility death equivalents/10 000 patient‐y)				−68.1 (−135.7 to −7.9)	
Probability of benefits outweigh risks§				98.7%	

ALI indicates acute limb ischemia; ECOD, efficacy cutoff date; ICH, intracranial hemorrhage; ITT, intent‐to‐treat; MCDA, multi‐criteria decision analysis; MI, myocardial infarction; and TIMI, Thrombolysis in Myocardial Infarction.

* End points are sorted by mean weight (1 – utility) from Table S3.

^†^ The 95% CIs of these rate differences were estimated using the Wald method (Liu 2006).

^‡^ Contributions shown are the average over the Monte Carlo simulations (see Data S1). Theoretical contributions are rate difference × mean weight.

^§^ Based on Monte Carlo simulations, accounting for statistical uncertainty of event rates and distribution of weights.

^‖^ Based on equation 2 (see Data S1).

**Figure 5:**
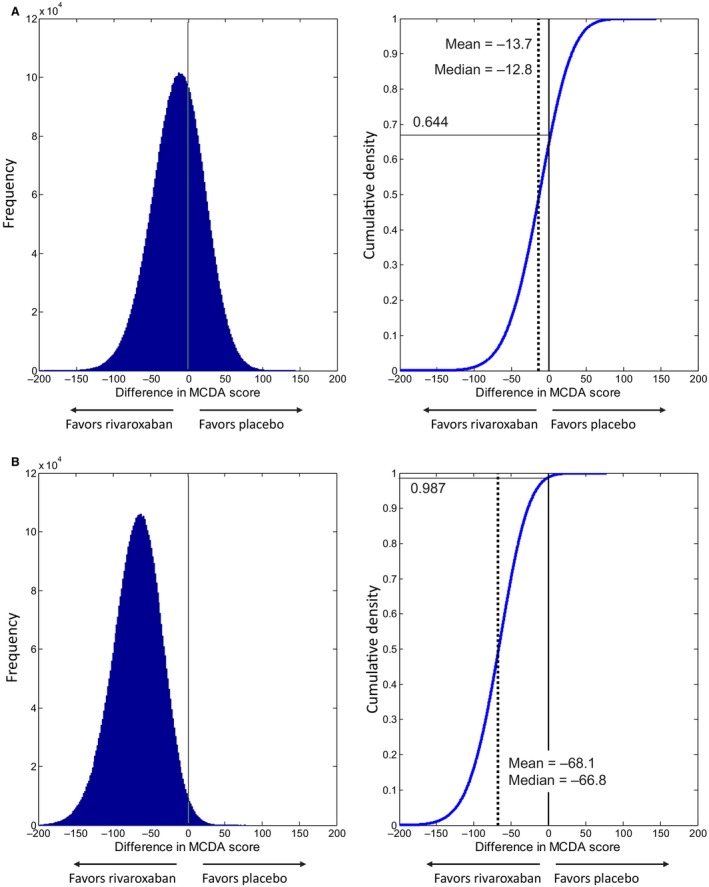
Probability density and cumulative density functions of the difference in multi‐criteria decision analysis (MCDA) scores based on uncertainty in weights and rates in the model 1 (A) intent‐to‐treat/until efficacy cutoff date and (B) safety/on‐treatment analyses. Left panel: distribution of the difference in MCDA scores based on Monte Carlo simulation, accounting for statistical uncertainty of both event rates and weights. Scores below 0 reflect benefits outweighing risks in favor of rivaroxaban. Right panel: cumulative density function for the difference in MCDA scores. The dotted line shows the mean and median. The cumulative density function value for an MCDA score difference equal to 0, 0.644 in (**A**) and 0.987 in (**B**), is the probability that benefits outweigh risks for rivaroxaban vs placebo.

The Monte Carlo simulation provided a cumulative density function of the difference in MCDA score with a probability of 64.4% that benefits outweigh risks favoring rivaroxaban plus aspirin in the ITT analysis (Figure 5). Similarly, for the on‐treatment analysis, treatment with rivaroxaban plus aspirin resulted in the utility equivalent of 68.1 (95% CI, 7.9–135.7) fewer deaths per 10 000 patient‐years compared with placebo plus aspirin, with the 95% CI excluding neutrality. The contributions to the MCDA score difference favored the rivaroxaban plus aspirin treatment arm for all of the end points except for nonfatal, nonintracranial hemorrhage TIMI major bleeding (Table 1). The cumulative density function of the difference in MCDA score gives a probability of 98.7% that benefits outweigh risks favoring rivaroxaban plus aspirin (Figure 5).

**Figure 6:**
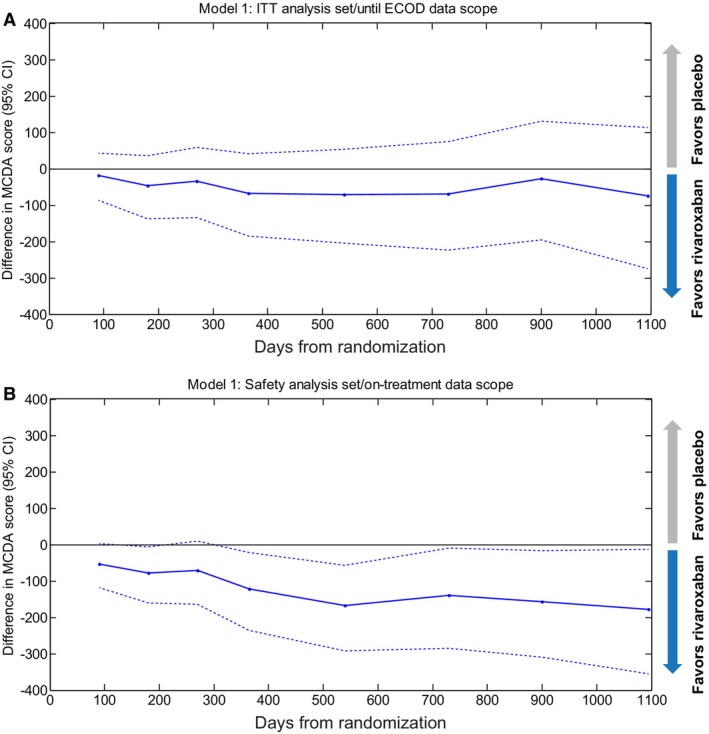
Benefit–risk over time in the multi‐criteria decision analysis (MCDA) model 1 analyses. (**A**) Intent‐to‐treat (ITT)/until efficacy cutoff date (ECOD) and (**B**) safety/on‐treatment. Lines show point estimates and 95% CIs for the between‐treatment difference in MCDA scores. Negative values favor rivaroxaban.

Finally, we assessed the temporal course of B‐R with the MCDA model using KM rates with a regular 3‐month time interval starting from approximately day 90 to day 1100 (Figure 6). It was noted that the temporal trend was more pronounced in the on‐treatment analyses than the ITT analyses, favoring rivaroxaban.

## Discussion

The VOYAGER PAD study was the second randomized trial that investigated the efficacy and safety of rivaroxaban in a clinical setting of patients with PAD. Despite currently available therapies, patients with symptomatic PAD who have undergone lower‐extremity revascularization remain at high risk for major adverse limb events and MACE, as shown in the placebo arm of the VOYAGER PAD study.^17^ Based on the unweighted B‐R analysis per 10 000 person‐years, the total reduction in the composite of the primary efficacy end point (ITT: 120 fewer events; on‐treatment: 181 fewer events) was greater than the TIMI major bleeding caused (ITT: 40 more events; on‐treatment: 29 more events) for rivaroxaban compared with placebo. Using an MCDA approach that takes into consideration the clinical importance of study end points (ie, weighted by HSUVs), rivaroxaban was associated with a greater reduction of utility death equivalents compared with placebo (ITT: 13.7; on‐treatment: 68.1, per 10 000 patient‐years). These findings suggest an overall favorable B‐R profile of rivaroxaban therapy.

While the notion that “the benefit must outweigh the risk” serves as a fundamental basis for regulatory agencies to approve a medical product, there is no one standard methodology to quantitate B‐R assessment. However, MCDA is one of several methods that has been increasingly used in B‐R assessment, particularly because of how it naturally integrates weighting. Generally, a wide spectrum of study end points may represent different clinical importance from the perspective of patients and physicians. The concept of fatal and irreversible harm related to antithrombotic therapy (eg, myocardial infarction, ischemic stroke, intracranial hemorrhage, cardiovascular death) was previously discussed by the FDA^19^, ^20^ and was used for the B‐R assessment in some studies without weighting.^22^ Interestingly, some researchers exercised the idea by applying empirical weight to various end points to assess the “net clinical benefit.”^28^, ^29^ In our weighted analysis, an MCDA approach was used, in which weights were extracted from the Tufts‐CEVR CEA registry by the independent research organization, Tufts‐CEVR. The search strategies were comprehensive, and the identified publications were relatively robust, taking into consideration health state definitions and similarities to the VOYAGER PAD trial in those published articles.

The results of unweighted B‐R assessment and weighted evaluation using an MCDA approach were generally favorable and consistent between the ITT and on‐treatment analyses. These findings are complementary to the observations in the first rivaroxaban study of this setting (ie, the COMPASS trial), which showed similar reductions in ischemic risk, including myocardial infarction, ischemic stroke, cardiovascular death, ALI, and major amputation of a vascular cause,^14^, ^30^ particularly in a subpopulation of patients with PAD. In addition, there were no significant differences in fatal bleeding, intracranial bleeding, or symptomatic bleeding into a critical organ between the rivaroxaban and placebo groups of the COMPASS trial.^14^, ^30^ However, it was noted that, for the VOYAGER PAD study, a favorable B‐R profile was less pronounced in the ITT analysis, which was driven in part by a nominal excess of death events in the rivaroxaban arm. The reason for this observation is not clearly understood given that these end points were nominally in favor of rivaroxaban therapy based on the analysis of events occurring while patients were on‐treatment (cardiovascular death: HR, 0.92 [95% CI, 0.68–1.25]; all‐cause death: HR, 0.84 [95% CI, 0.64–1.09]). The impact of permanently discontinuing study medication (≈14% per year) in the VOYAGER PAD trial has been previously described.^17^ In addition, in the COMPASS trial subpopulation of patients with PAD, rivaroxaban was associated with nominal reductions in cardiovascular and all‐cause death compared with placebo in an ITT analysis (Table S7). A meta‐analysis of COMPASS and VOYAGER PAD demonstrated a clear benefit for MACE with relatively neutral results on mortality.^14^ Moreover, it was observed that rivaroxaban at the same low dose was associated with a reduction in cardiovascular and all‐cause death in ATLAS II (An Efficacy and Safety Study for Rivaroxaban in Patients With Acute Coronary Syndrome)^31^ and showed no increase in either of these end points in the COMMANDER HF (A Study to Assess the Effectiveness and Safety of Rivaroxaban in Reducing the Risk of Death, Myocardial Infarction or Stroke in Participants With Heart Failure and Coronary Artery Disease Following an Episode of Decompensated Heart Failure) trial.^32^ Given the totality of the data, the nominal excess of death events that occurred following permanent discontinuation of treatment in the rivaroxaban arm is more likely a chance finding of the study than a signal of harm of rivaroxaban treatment in this patient population. Finally, our 1‐way sensitivity MCDA analysis suggested that the overall B‐R balance could be sensitive to the utility value associated with ALI. These data could at first suggest that the treatment benefit associated with rivaroxaban might tend to be neutral or diminished, if one would consider ALI to be of limited clinical importance. However, prior research had shown that the short‐term risk of all‐cause mortality and major amputation is especially elevated following ALI.^33^

Limitations of our MCDA approach include: (1) assumption of independence among different types of events and the assumption that the additive model underlying the MCDA is representative of the tradeoffs patients or physicians make in anticoagulant treatment; (2) the use of HSUV values for a mix of chronic and acute events (generally, HSUV values are using quality‐adjusted life‐year–type calculations, in which the HSUVs are integrated over time with that corresponding health state); (3) the use of a literature search for the HSUVs assessed in different studies and with different methods, rather than a dedicated study to assess preference weights specific to PAD with revascularization; and (4) a simplified version of the canonical MCDA formulation, in which we used linear value functions and did not impose any constraints on the end point ranges over which the value function applies. The authors regard these assumptions as impacting the specific quantitative values obtained but not their interpretation or the B‐R assessment.

In summary, in patients with PAD who had undergone lower‐extremity revascularization receiving antiplatelet therapy, rivaroxaban 2.5 mg twice daily demonstrated a favorable B‐R profile compared with placebo, and the findings were more pronounced with the on‐treatment analysis and generally consistent between unweighted assessment and weighted MCDA analyses.

## Sources of Funding

This analysis was supported by Janssen Research & Development, LLC.

## Disclosures

Drs Yuan, Levitan, Haskell, and Barnathan, and H. Deng are full‐time employees of Janssen Research & Development, LLC, and own stock and stock options in Johnson & Johnson. The remaining authors have no disclosures to report.

## Supporting information

Data S1
