# Supplementary material for: Quantitative Benefit–Risk Evaluation of Rivaroxaban in Patients After Peripheral Arterial Revascularization: The VOYAGER PAD Trial
Source: J Am Heart Assoc. 2024 Apr 2;13(8):e032782. doi: 10.1161/JAHA.123.032782 (PMC11262494; doi:10.1161/JAHA.123.032782)
Supplement: Supplementary file 1 — Data S1 [file JAH3-13-e032782-s001.pdf]

SUPPLEMENTAL MATERIAL

Data S1. MCDA Methodological Details

Table S1. Endpoints in two MCDA models

Model 1 Endpoints	Model 2 Endpoints
• Composite of CV death and fatal bleeding<br>• Non-fatal major amputation of vascular etiology<br>• Non-fatal ALI<br>• Non-fatal ischemic stroke<br>• Non-fatal MI<br>• Non-fatal intracranial TIMI major bleeding<br>• Non-fatal, non-intracranial TIMI major bleeding	• All-cause death<br>• Non-fatal major amputation of vascular etiology<br>• Non-fatal ALI<br>• Non-fatal ischemic stroke<br>• Non-fatal MI<br>• Non-fatal intracranial TIMI major bleeding<br>• Non-fatal, non-intracranial TIMI major bleeding

ALI, acute limb ischemia; CV Death, death from cardiovascular causes; MCDA, multiple-criteria decision analysis; MI, myocardial infarction; TIMI, Thrombolysis in Myocardial Infarction.

Table S2. Summary of Health State Utilities for 6 Health States From Literature Review

	Major Amputation (n=6)	ALI (n=52)	MI (n=48)	Ischemic Stroke (n=45)	Intracranial Bleeding (n=5)	Major non-intracranial Bleeding (n=3)
**Mean (SD)**	0.41 (0.13)	0.64 (0.17)	0.78 (0.13)	0.65 (0.19)	0.58 (0.24)	0.78 (0.11)
**Median (IQR)**	0.44 (0.33–0.50)	0.64 (0.60–-0.74)	0.79 (0.72–0.87)	0.70 (0.63–0.75)	0.65 (0.62–0.70)	0.84 (0.75–0.84)

ALI, acute limb ischemia; IQR, interquartile range; MI, myocardial infarction; SD, standard deviation.
Note: The lower health-related quality of life of a particular health state, the higher severity ranking.

Mathematical Implementation of Utility Weights in the MCDA

The simulation was developed in MATLAB Version 7.11.0.584 (R2010b) using the Statistics Toolbox Version 7.4 (R2010b) (MathWorks, Natick, MA, USA).

The standard approach in multiple-criteria decision analysis (MCDA) uses mathematical formulations called value functions to support attributes that can be measured in any form (eg, proportions, incidence rates, time spans, categorical outcomes). The value functions serve 2 purposes: First, they convert any attribute form into values on a normalized 0 to 1 scale that can then be multiplied by weights. Second, they accommodate varying returns to scale, allowing the impact of a given change in the attribute to vary depending on the particular starting point of that change (e.g., the impact on a decision of a change in probability of death

from 0% to 5% will generally be considered much larger than that for a change from 50% to 55%). In our case, since all endpoints in the value tree are time-to-first-event endpoints measured with incidence rates or Kaplan-Meier rates, we can avoid converting the attributes to a normalized scale. Additionally, the endpoint values do not span a large range for each endpoint, so we use linear returns to scale. For these reasons, we used an MCDA model with linear value functions, where the attributes were the incidence rates or Kaplan-Meier estimates for each endpoint. With this approach, the MCDA score for each treatment in VOYAGER PAD reduces to:

$$S^j = \sum_{i=1}^{N} w_i r_i^j \qquad \text{(equation 1)}$$

Where S^j is the MCDA score for treatment *j*, *j* is the VOYAGER PAD arm (rivaroxaban + aspirin or placebo + aspirin), *N* is the number of endpoints in the MCDA, w_i is the weight for endpoint *i*, and r_i^j is the rate for endpoint *i* in treatment *j*. The contribution of endpoint *i* to the MCDA score for arm *j* is $w_i r_i^j$.

The difference in MCDA score between rivaroxaban + aspirin and placebo + aspirin can be expressed as:

$$MCDA\ score\ difference = \sum_{i=1}^{N} w_i (r_i^{Riva} - r_i^{PBO}) = \sum_{i=1}^{N} w_i RD_i \qquad \text{(equation 2)}$$

Where $RD_i = r_i^{Riva} - r_i^{PBO}$ is the rate difference between the treatments for endpoint *i*. The 95% confidence intervals (CIs) of these rate differences are estimated using the Wald method (Liu 2006) assuming normal approximation.

In addition to the point estimate from the MCDA model, we assess results probabilistically using Monte Carlo simulations that account for all uncertainty in the rates and the weights, an approach referred to as stochastic multi-attribute acceptability analysis. To account for uncertainty in event rates, the number of events (X_i^j for outcome *i* and treatment arm *j*) is randomly drawn from a Poisson distribution for each outcome, treatment arm, and data scope:

$$X_i^j \sim Poisson(\lambda_i^j)$$

where $\hat{\lambda}_i^j = n_i^j$, n_i^j is the number of patients with observed events for endpoint *i* in VOYAGER PAD treatment arm *j* during the observation period for a given data scope.

For these simulations, the clinical trial data are presented in Table 1. The weights for the simulations are given in Table S3 and the relevant simulation parameters are listed in Table S4. For each round of the Monte Carlo simulation, the rate difference for endpoint *i* is calculated as:

$$RD_i = \frac{X_i^{Riva}}{exposure\ time_i^{Riva}} - \frac{X_i^{PBO}}{exposure\ time_i^{PBO}}$$

where $exposure\ time_i^j$ is the cumulative exposure time for endpoint *i* in VOYAGER PAD treatment arm *j* for that same data scope.

Table S3: Utilities Weights and Their Standard Deviations

Endpoint	Distribution	Utility mean	Weight (1 - utility)	Standard deviation
Composite of CV death and fatal TIMI major bleeding	NA	0	1.00	0
Non-fatal major amputation	Beta	0.41	0.59	0.13
Non-fatal intracranial TIMI major bleeding	Beta	0.58	0.42	0.24
Non-fatal ALI	Beta	0.64	0.36	0.17
Non-fatal ischemic stroke	Beta	0.65	0.35	0.19
Non-fatal MI	Beta	0.78	0.22	0.13
Non-fatal, non-intracranial TIMI major bleeding	Beta	0.78	0.22	0.11

ALI, acute limb ischemia; CV Death, death from cardiovascular causes; MI, myocardial infarction; N/A, not available; TIMI, Thrombolysis in Myocardial Infarction.

Table S4. Relevant Parameters Used in the Monte Carlo Simulations

Data scope/endpoint	Rivaroxaban		Placebo	
	n (# of patients with events)	Exposure time (PYs)	*n* (# of patients with events)	Exposure time (PYs)
ITT/Until ECOD				
Composite of CV Death and fatal TIMI major bleeding	211	7,987.6	182	7,993.6
Non-fatal major amputation	94	7,798.6	111	7,790.9
Non-fatal intracranial TIMI major bleeding	18	7,919.3	16	7,926.0
Non-fatal ALI	147	7,696.3	220	7,553.2
Non-fatal ischemic stroke	60	7,860.7	71	7,853.5
Non-fatal MI	110	7,802.0	120	7,776.6
Non-fatal, non-intracranial TIMI major bleeding	61	7,862.3	41	7,894.0
Safety/On-treatment				
Composite of CV Death and fatal TIMI major bleeding	83	6,521.0	92	6,635.6
Non-fatal major amputation	48	6,449.4	65	6,531.9
Non-fatal intracranial TIMI major bleeding	11	6,503.6	13	6,613.5
Non-fatal ALI	121	6,383.2	188	6,401.4
Non-fatal ischemic stroke	45	6,481.6	53	6,587.1
Non-fatal MI	73	6,458.6	93	6,551.8
Non-fatal, non-intracranial TIMI major bleeding	45	6,480.9	25	6,595.6

ALI, acute limb ischemia; CV Death, death from cardiovascular causes; ECOD, efficacy cutoff date; ITT, intention-to-treat; MI, myocardial infarction; PY, patient-year; TIMI, Thrombolysis in Myocardial Infarction.

The weights w_i in the MCDA were based on a health state utility literature review conducted by the Tufts Center for the Evaluation of Value and Risk in Health (Tufts-CEVR). The Tufts-CEVR Cost-Effectiveness Analysis (CEA) Registry is a database of over 9,080 cost-utility analyses and 33,442 health state utility values (HSUVs). HSUVs reflect the level of health and function associated with a given health state. Perfect health equates a utility value of 1, whereas death equates a utility value of zero. The literature review provided mean and standard deviation of utilities (Table S3).

To account for the uncertainty in the weight for endpoints in Table S3, the HSUVs were assumed to follow beta distributions in the Monte Carlo simulations. Weights in the MCDA models were defined as 1 – HSUV value, so death has a weight of 1, enabling a straightforward interpretation of the between treatment difference in MCDA score as the utility-equivalent of the difference in the number of deaths between treatments. Alpha and beta parameters for the beta distributions were computed from the mean and standard deviations of utilities from the literature review.

To estimate a 95% CI based on uncertainty in both the clinical data and the weights, the Monte Carlo simulation was used to generate a cumulative density function of the between-treatment difference in MCDA score (Figure 4). The 95% CI was based on the location of the 2.5% and 97.5% percentiles using 5,000,000 simulation runs. The probability that benefits outweigh risks is the value of the cumulative density function for a zero difference in MCDA score (rivaroxaban – placebo) over all simulations.

Table S5: Baseline Characteristics of the Patients in the VOYAGER PAD Trial[17,*]

Characteristic	Rivaroxaban (N = 3286)	Placebo (N = 3278)
Median age (IQR) — yr	67.0 (61.0–73.0)	67.0 (61.0–73.0)
Female sex — no. (%)	847 (25.8)	857 (26.1)
Median body-mass index (IQR)†	26.0 (23.3–29.1)	26.0 (23.2–29.1)
Race — no. (%)‡		
White	2647 (80.6)	2656 (81.0)
Asian	484 (14.7)	482 (14.7)
Black	84 (2.6)	71 (2.2)
Other	71 (2.2)	69 (2.1)
Geographic region — no. (%)		
North America	347 (10.6)	347 (10.6)
Western Europe	914 (27.8)	912 (27.8)
Eastern Europe	1301 (39.6)	1298 (39.6)
Asia Pacific	481 (14.6)	480 (14.6)
South America	243 (7.4)	241 (7.4)
Risk factors and coexisting conditions — no. (%)		
Hypertension	2684 (81.7)	2658 (81.1)
Hyperlipidemia	1971 (60.0)	1968 (60.0)
Current smoker	1147 (34.9)	1132 (34.5)
Diabetes mellitus	1313 (40.0)	1316 (40.1)
Estimated GFR <60 ml/min/1.73 m^2	661 (20.1)	666 (20.3)
Symptomatic coronary artery disease	1052 (32.0)	1015 (31.0)
Myocardial infarction	365 (11.1)	349 (10.6)
Known carotid artery disease	282 (8.6)	293 (8.9)
Peripheral artery disease–related history		
Median ankle–brachial index (IQR)	0.56 (0.42–0.67)	0.56 (0.42–0.67)
Previous amputation — no. (%)	194 (5.9)	196 (6.0)
History of claudication — no. (%)	3132 (95.3)	3137 (95.7)
History of critical limb ischemia — no. (%)	999 (30.4)	969 (29.6)
Previous peripheral revascularization — no. (%)	1181 (35.9)	1155 (35.2)
Qualifying revascularization — no. (%)		
Performed for claudication	2521 (76.7)	2504 (76.4)
Performed for critical limb ischemia§	762 (23.2)	771 (23.5)
Endovascular	2153 (65.5)	2140 (65.3)
Surgical	1133 (34.5)	1138 (34.7)
Medications — no. (%)		
Statin	2608 (79.4)	2641 (80.6)
ACE inhibitor or ARB	2096 (63.8)	2063 (62.9)
Aspirin at randomization	3256 (99.1)	3248 (99.1)
Clopidogrel at randomization	1658 (50.5)	1655 (50.5)

*There were no significant differences between groups. Percentages may not total 100 because of rounding. ACE denotes angiotensin-converting enzyme, ARB angiotensin-receptor blocker, GFR glomerular filtration rate, and IQR inter- quartile range.
†The body-mass index is the weight in kilograms divided by the square of the height in meters.
‡Race was reported by the patient.
§Critical limb ischemia was defined as patients with chronic ischemic rest pain, ulcers, or gangrene attributable to objectively proven arterial occlusive disease. Hemodynamics: rest pain with ankle pressure ≤50 mmHg and toe pressure ≤30 mmHg or tissue loss (ulcer or gangrene) ≤70 mmHg and toe pressure ≤50 mmHg.

From the *New England Journal of Medicine*, Bonaca MP, Bauersachs RM, Anand SS, et al. Rivaroxaban in Peripheral Artery Disease After Revascularization, volume 382, pages 1994-2004. Copyright © 2020 Massachusetts Medical Society. Reprinted with permission from Massachusetts Medical Society.

Table S6: Number needed to treat and number needed to harm* for key efficacy and safety endpoints (rivaroxaban 2.5 mg bid + aspirin 100 mg od vs placebo bid + aspirin 100 mg od) in the ITT/Until ECOD and Safety/On-treatment populations for all patients and age subgroups: the VOYAGER study

Study endpoint	ITT/Until ECOD Population	Safety/On-treatment Population
Primary efficacy outcome – all patients	–83	–55
<65 years	–222	–100
65–75 years	–60	–45
>75 years	–56	–36
Myocardial infarction – all patients	–455	–283
<65 years	–227	–233
65–75 years	–417	–172
>75 years	303	10,000
Ischemic stroke - all patients	–714	–526
<65 years	–667	–435
65–75 years	–3333	–625
>75 years	–294	–833
Cardiovascular death – all patients	323	–1000
<65 years	714	–476
65–75 years	217	–3333
>75 years	238	10,000
Acute limb ischemia – all patients	–101	–91
<65 years	–250	–208
65–75 years	–85	–79
>75 years	–53	–45
Major amputation of vascular etiology – all patients	–625	–500
<65 years	500	3333
65–75 years	–435	–357
>75 years	–133	–182
TIMI major bleeding (CABG or non-CABG) – all patients	250	345
<65 years	435	1429
65–75 years	147	164
>75 years	500	714
Fatal bleeding – all patients	909	NA
<65 years	714	1429
65–75 years	909	–3333
>75 years	1667	–1250
Non-fatal intracranial bleeding – all patients	3333	–3333
<65 years	2000	–2500
65–75 years	588	588
>75 years	–303	–250
Other non-fatal non-ICH TIMI major bleedings – all patients	385	313
<65 years	3333	2000
65–75 years	250	213
>75 years	217	159

*NNT/NNH values are the reciprocal of the rate difference. Negative values favor rivaroxaban while positive values favor placebo.

Table S7: Efficacy Results in VOYAGER PAD (Intent-to-Treat Population) and COMPASS subpopulation with PAD

	VOYAGER			COMPASS PAD		
	XARELTO N=3286	Placebo N=3278	Hazard Ratio (95% CI)[*] p-value[†]	XARELTO N=2492	Placebo N=2504	Hazard Ratio (95% CI)[*]
Outcome Components	Event Rate (%/year)			Event Rate (%/year)		
5-Component Outcome (Major thrombotic vascular events)[‡]	6.8	8.0	0.85 (0.76, 0.96) p=0.0085	3.4	4.8	0.71 (0.57, 0.87)
MI	1.7	1.9	0.88 (0.70, 1.12)	1.1	1.5	0.76 (0.53, 1.09)
Ischemic Stroke[§]	0.9	1.0	0.87 (0.63, 1.19)	0.5	0.9	0.55 (0.33, 0.93)
CV death[¶]	2.5	2.2	1.14 (0.93, 1.40)	1.4	1.7	0.82 (0.59, 1.14)
ALI	2.0	3.0	0.67 (0.55, 0.82)	0.4	0.8	0.56 (0.32, 0.99)
Major amputation of a vascular etiology[#]	1.3	1.5	0.89 (0.68, 1.16)	0.2	0.6	0.40 (0.20, 0.79)
VOYAGER Secondary Efficacy Outcomes[Þ]						
MI, ischemic stroke, CHD death,[ß] ALI, and major amputation due to vascular etiology	5.8	7.3	0.80 (0.71, 0.91) p=0.0008	2.8	4.2	0.66 (0.53, 0.83)
Unplanned index limb revascularization for recurrent limb ischemia[à]	8.4	9.5	0.88 (0.79, 0.99) p=0.028	N/A	N/A	N/A
Hospitalization for a coronary or peripheral cause of a thrombotic nature[#]	3.5	4.8	0.72 (0.62, 0.85) p<0.0001	1.7	2.9	0.58 (0.44, 0.77)
MI, ischemic stroke, all-cause mortality, ALI, and major amputation due to vascular etiology	8.2	9.3	0.89 (0.79, 0.99) p=0.029	4.8	6.0	0.80 (0.67, 0.96)
MI, all-cause stroke, CV death, ALI, and major amputation due to vascular etiology	6.9	8.1	0.86 (0.76, 0.96) p=0.010	3.4	4.9	0.70 (0.57, 0.86)
All-cause mortality	4.0	3.7	1.08 (0.92, 1.27)	2.8	3.1	0.91 (0.72, 1.16)
VTE events[è]	0.3	0.5	0.61 (0.37, 1.00)	0.2	0.3	0.67 (0.30, 1.49)

Efficacy endpoints in COMPASS PAD were analysed according to the pre-specified endpoints in VOYAGER when applicable.

* XARELTO vs. placebo.

† Two-sided p-values

‡ Major thrombotic vascular event is the composite of MI, ischemic stroke, CV death, ALI, and major amputation of a vascular etiology.

§ Ischemic stroke for VOYAGER included stroke of uncertain/unknown etiology whereas COMPASS only included ischemic stroke.

¶ CV death includes Coronary Heart Disease death, or death due to other CV causes or sudden cardiac arrest and unknown death.

\# Adjudicated events in VOYAGER and investigator reported events in COMPASS

Þ Secondary outcomes for VOYAGER were tested sequentially.

ß CHD death includes death due to sudden cardiac death, MI, or coronary revascularization procedure

à Unplanned index limb revascularization for recurrent limb ischemia was not captured in COMPASS study.

è Investigator reported in VOYAGER and adjudicated events in COMPASS

ALI=acute limb ischemia, CHD=coronary heart disease; CI=confidence interval, CV=cardiovascular; MI=myocardial infarction, VTE=venous thromboembolism.
